# Long-Term Glycemic Variability Is Associated With Arterial Stiffness in Chinese Adults

**DOI:** 10.3389/fendo.2021.711540

**Published:** 2021-09-16

**Authors:** Yuwen Zhang, Shujing Wu, Mian Li, Tiange Wang, Min Xu, Jieli Lu, Shuangyuan Wang, Jie Zhang, Yufang Bi, Weiqing Wang, Guang Ning, Yu Xu, Yuhong Chen

**Affiliations:** ^1^Department of Endocrine and Metabolic Diseases, Shanghai Institute of Endocrine and Metabolic Diseases, Ruijin Hospital, Shanghai Jiao Tong University School of Medicine, Shanghai, China; ^2^Shanghai National Clinical Research Center for Metabolic Diseases, Key Laboratory for Endocrine and Metabolic Diseases of the National Health Commission of the PR China, Shanghai National Center for Translational Medicine, Ruijin Hospital, Shanghai Jiao Tong University School of Medicine, Shanghai, China

**Keywords:** glycemic variability, brachial-ankle pulse wave velocity, arterial stiffness, subclinical atherosclerosis, cardiovascular disease

## Abstract

**Objective:**

The aim of the study was to investigate the association between the visit-to-visit variability (VVV) of fasting plasma glucose (FPG) and arterial stiffness in Chinese adults.

**Methods:**

We performed a cohort study involving 2002 Chinese adults with no history of myocardial infarction or stroke. All the participants attended three visits (the baseline visit in 2008, the 2^nd^ visit in 2009 and the 3^rd^ visit in 2013). We used four measures to define the VVV of FPG across the three visits: the standard deviation (SD), the coefficient of variation (CV), the average successive variability (ASV) and the variability independent of the mean (VIM). We used brachial-ankle pulse wave velocity (ba-PWV) to measure arterial stiffness at the 2^nd^ and the 3^rd^ visits.

**Results:**

Compared with the lowest tertile of all the four measurements of VVV of FPG, significantly increased levels of ba-PWV change, ratio of ba-PWV change and the occurrence of the elevated ba-PWV were found in the highest tertile. The odds ratio (OR) and 95% confidence interval (CI) comparing participants in the highest tertile *vs*. the lowest tertile of FPG-SD was 1.37 (1.01-1.86) for risks of having elevated ba-PWV, even after adjustment for covariates including the mean FPG. Similar results were found for FPG-CV and FPG-VIM.

**Conclusion:**

Greater long-term variability of FPG was associated with an increased risk of arterial stiffness, suggesting that the VVV of FPG could be used for an early detection of subclinical atherosclerosis.

## Introduction

Glycemic impairment even within the nondiabetic range has been considered to be an independent risk factor for cardiovascular disease (CVD) (1–4). Previous studies investigating the hyperglycemia-related complications have mainly relied on blood glucose assessment at a single point in time, which may not capture the true underlying glucose levels over time, while glycemic variability is recognized recently as a measure that could more accurately capture the pathological processes before the occurrence of complications.

Glycemic variability is an assessment of changes in glycemia of both short and long terms. The changes of glycemia in one day or between two days are short-term glycemic variability, which can be evaluated through continuous glucose monitoring (CGM) (5). If the changes are within months or years, they are regarded as long-term glycemic variability, which can be evaluated through visit-to-visit variability of hemoglobin A1c (HbA1c) or blood glucose (5). Previous studies on glycemic variability were mainly conducted in people with diabetes and focused on short-term variability (6), the prognostic value of long-term glycemic variability has been understudied in the general population.

Arterial stiffness is regarded as a precursor of clinical CVD (7, 8). The brachial-ankle pulse wave velocity (ba-PWV) measures arterial stiffness in a simple and noninvasive way (9). The ba-PWV has been reported to play an important role in predicting CVD events among the general population (10) and patients with diabetes (11, 12). Previous studies mainly examined the associations between glycemic variability and CVD development. Investigations on the associations of long-term glycemic variability with subclinical atherosclerosis, such as arterial stiffness in a general population are rare. Therefore, we aimed to investigate whether the visit-to-visit variability (VVV) of fasting plasma glucose (FPG) independently associated with arterial stiffness in Chinese adults aged 40 years or above.

## Materials And Methods

### Study Population

**Figure 1:**
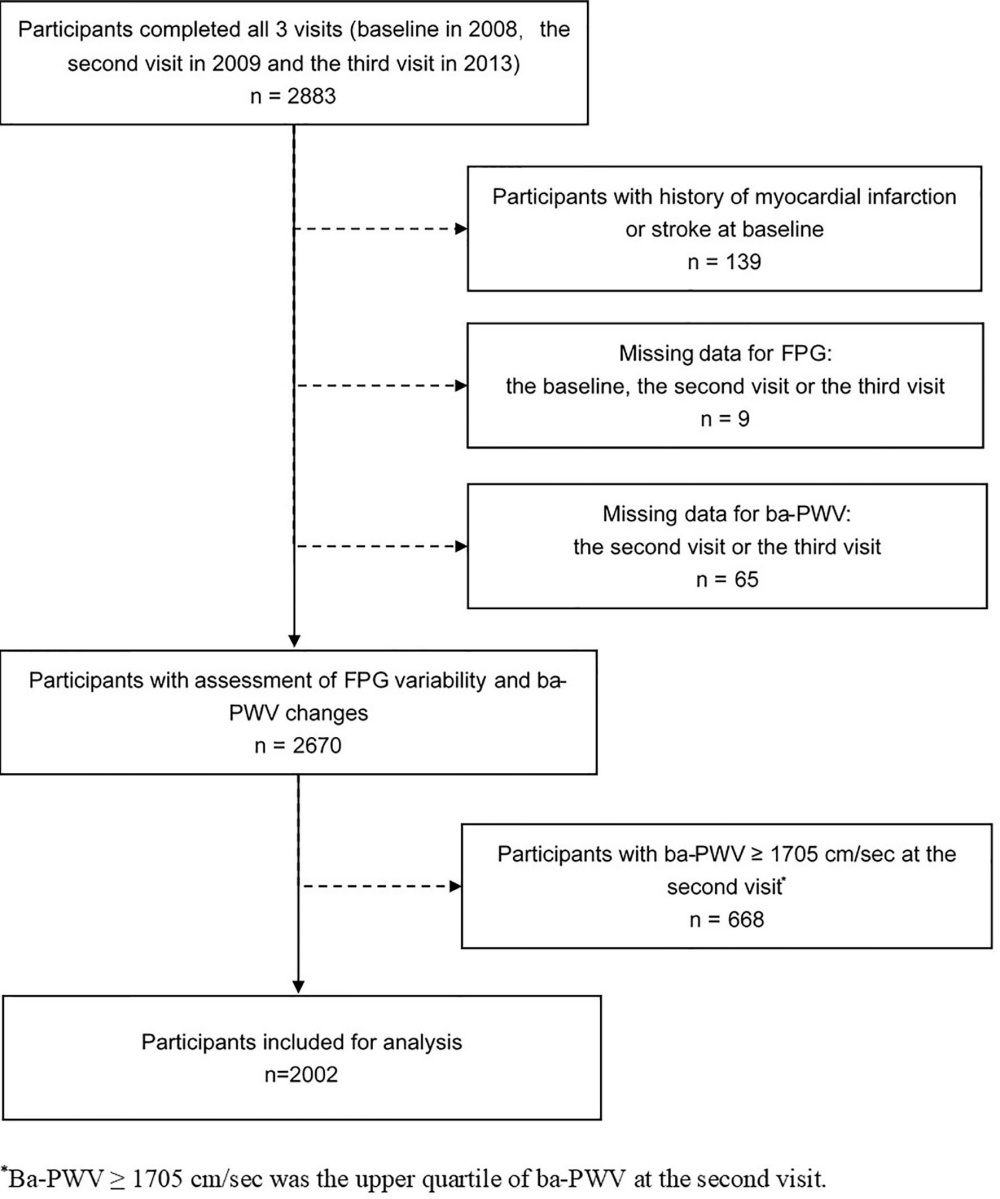
Flow chart of the study population.

The study was conducted in a community-based population in Songnan Community, Baoshan District, Shanghai, China, as reported previously (13, 14). The design of the current study has also been published previously (15). In brief, a total of 2883 permanent residents aged 40 years or above in a local community were enrolled and underwent 3 examination visits (the baseline, the 2^nd^ and the 3^rd^ visits in June through July 2008, June through August 2009 and March through May 2013. At the baseline visit, the participant underwent a screening examination including a FPG measurement. In the 2^nd^ and the 3^rd^ visits, all the participants underwent a comprehensive examination including an FPG measurement and an evaluation of arterial stiffness using ba-PWV. After excluding individuals with a history of myocardial infarction or stroke prior to the baseline visit (n = 139), those with missing data for FPG at any of the 3 visits (n = 9), those with missing data for the 2^nd^ visit or the 3^rd^ visit ba-PWV (n = 65), and those with the 2^nd^ visit ba-PWV levels within the upper quartile (≥ 1705 cm/sec) (n = 668), 2002 participants (771 men and 1231 women) were finally included in the current analysis (**Figure 1**).

The study protocol has been approved by the Institutional Review Board of Ruijin Hospital, Shanghai Jiao Tong University School of Medicine and all participants have provided written informed consent.

### Data Collection

All the participants provided demographic and lifestyle factors, previous medical history and medication use through a standard questionnaire administered by trained physicians at the 3 visits. Current smoking status was defined as yes if the subject smoked one cigarette per day or seven per week regularly during the past 6 months. Current drinking status was defined as yes if the subject consumed alcohol at least once per week regularly during the past 6 months (14). The information on participants’ physical activities was estimated using the International Physical Activity Questionnaire and the cut point of being physically active was the upper tertile of metabolic equivalent-hours per week like our previous study (15, 16). Body weight, height and waist circumference (WC) were measured according to a standard protocol. Body mass index (BMI) was calculated by dividing weight (in kilograms) by height (in meters) squared. After at least 5 minutes of rest, blood pressure (BP) was measured 3 times consecutively with one-minute intervals using an automated electronic device (OMRON Model HEM-752, Omron Company, Dalian, China). The average of the 3 measurements was used in analysis.

After an overnight fast (at least 10 h), venous blood samples were collected for laboratory measurements at the 3 visits. The FPG was measured by the glucose oxidase method on an autoanalyser (ADVIA-1650 Chemistry System, Bayer, Leverkusen, Germany). Serum concentrations of triglycerides (TG), total cholesterol (TC), high-density lipoprotein cholesterol (HDL-c) and low-density lipoprotein cholesterol (LDL-c) were determined by chemiluminescence method with the same autoanalyzer.

### Measures of Glycemic Variability

We calculated the VVV of FPG by using FPG levels at each of the 3 visits. Given that there are no internationally agreed upon standard measures of long-term glycemic variability, we opted to include a wide range of measures as our previous study on VVV of BP (15). We used the following measures to define the VVV of FPG: 1) the standard deviation (SD); 2) the coefficient of variation (CV), calculated by SD divided by mean; 3) the average successive variability (ASV), defined as the average absolute difference between successive values; 4) the variability independent of the mean (VIM), calculated as 100 * SD/mean^β^, where β is the regression coefficient based on natural logarithm of SD on natural logarithm of mean. All the four variables have been described in several other previous studies (5, 17–19).

### Measurement of ba-PWV

As reported previously (15, 20), the ba-PWV value was examined by Colin VP-1000 (Model BP203RPEII, form PWV/ABI; OMRON Colin Medical Instruments, Tokyo, Japan). After taking a 15-30 min rest, participants attached cuffs around both arms and ankles. The ba-PWV value was obtained automatically as the transit distance between the arm and ankle, which was calculated according to body height, divided by the transit time, which was defined as the time interval between the initial increase in brachial and tibial waveforms. The greater value of the right and the left ba-PWV was used for analysis (15).

The upper quartile of the 2^nd^ visit ba-PWV was defined as an elevated ba-PWV (≥ 1705 cm/sec). Ba-PWV change was calculated as the 3^rd^ visit ba-PWV-the 2^nd^ visit ba-PWV. The ratio of ba-PWV change was calculated as ba-PWV change/the 2^nd^ visit ba-PWV.

### Statistical Analysis

SPSS version 22.0 (SPSS Inc., Chicago, IL, USA) was used for database management and statistical analysis. Participants were categorized into three groups according to the tertiles of FPG-SD. Data are presented as means ± SD or medians (interquartile ranges) for continuous variables or numbers (percentages) for categorical variables. For continuous variables with skewed distribution such as TG, we log-transformed it before analysis. We compared general characteristics among these three groups using the ANOVA test for continuous variables and the χ^2^ tests for categorical variables.

To identify the associations of VVV of FPG with the ba-PWV changes, multiple linear regression analysis was used. We used 4 models to adjust for covariates: Model 1 was adjusted for age and sex; Model 2 was adjusted for the variables in model 1, plus education, current smoking, current drinking, physical activity, diabetes status, use of antidiabetic medications, use of statins and use of angiotensin converting enzyme inhibitors (ACEIs) or angiotensin receptor blockers (ARBs); Model 3 was adjusted for the variables in model 2, plus baseline WC, systolic BP, log_10_ TG, LDL-c, log_10_ (change of TG) and change of LDL-c. Changes of TG and LDL-c were the absolute differences of TG and LDL-c over the five years; Model 4 was adjusted for the variables in model 3, plus the average FPG and the 2^nd^ visit ba-PWV.

To estimate the odds ratios (ORs) of an elevated ba-PWV and VVV of FPG, the multivariate-adjusted logistic regression analysis was conducted. We used tertiles of each measures of FPG variability with the lowest tertile as the reference in 4 models: Model 1 was adjusted for age and sex; Model 2 was adjusted for the variables in model 1, plus education, current smoking, current drinking, physical activity, diabetes status, use of antidiabetic medications, use of statins and use of ACEIs or ARBs; Model 3 was adjusted for the variables in model 2, plus baseline WC, systolic BP, log_10_ TG, LDL-c, log_10_ (change of TG) and change of LDL-c; Model 4 was adjusted for the variables in model 3, plus the average FPG. The possible interaction between the diabetic status and VVV of FPG with elevated ba-PWV was tested by adding the interaction term into the model using the likelihood ratio test. The multivariate logistic regression analysis with forward stepwise (conditional) method was conducted to find out which one of the four measures of FPG variability was more closely related to an elevated ba-PWV.

All tests were two-tailed, with a *P* value<0.05 considered as statistically significant.

## Results

### Characteristics of Study Participants

The general demographic and clinical characteristics of 2002 study participants were summarized in **Table 1**. Overall, the mean age was 55.8 ± 8.3 years and 38.5% (n = 771) were males. With the increase of FPG-SD tertiles, participants were more likely to be older, to have diabetes or hypertension, to have higher levels of BMI, WC, BP, TG, TC, and FPG, and with a higher proportion of antidiabetic drug use.

**Table 1:** Characteristics of study participants by tertiles of FPG-SD.

Characteristics	Overall	T1 (0-6.5 mg/dL)	T2 (6.5-11.9 mg/dL)	T3 (11.9-114.2 mg/dL)	*P-*value
Participants, n	2,002	666	667	669	/
FPG-SD, mg/dL	8.8 (5.5-15.2)	4.5 (3.2-5.5)	8.8 (7.6-10.1)	20.7 (15.2-34.4)	/
Age, years	55.8 ± 8.3	55.6 ± 8.1	55.3 ± 8.4	56.7 ± 8.4	0.007
Men, n (%)	771 (38.5)	219 (32.9)	253 (37.9)	299 (44.7)	<0.001
Body mass index, kg/m^2^	25.2 ± 3.7	24.7 ± 3.4	25.0 ± 3.9	26.1 ± 3.8	<0.001
Waist circumference, cm	84.5 ± 10.0	82.7 ± 9.3	83.6 ± 10.0	87.2 ± 10.1	<0.001
High school education or above, n (%)	592 (29.6)	204 (30.6)	208 (31.2)	180 (26.9)	0.176
Life style factors, n (%)					
Current smoking	517 (25.8)	161 (24.2)	162 (24.3)	194 (29.0)	0.071
Current drinking	349 (17.4)	110 (16.5)	118 (17.7)	121 (18.1)	0.734
Physically active	685 (34.2)	240 (36.0)	236 (35.4)	209 (31.2)	0.134
Blood pressure, mmHg					
Systolic blood pressure,	128.1 ± 18.7	125.4 ± 18.2	126.9 ± 18.0	132.1 ± 19.1	<0.001
Diastolic blood pressure	78.8 ± 10.2	77.6 ± 10.1	78.2 ± 10.1	80.6 ± 10.3	<0.001
Fasting plasma glucose, mg/dL	101.1 ± 33.5	90.2 ± 12.3	89.6 ± 14.9	123.4 ± 47.3	<0.001
Average fasting plasma glucose, mg/dL	105.5 ± 30.4	92.4 ± 12.1	94.6 ± 14.0	129.3 ± 39.7	<0.001
Lipid profile, mg/dL					
Triglycerides	124.0 (87.7-181.6)	116.0 (82.4-159.9)	119.6 (84.1-175.4)	139.0 (97.0-209.0)	<0.001
Total cholesterol	197.1 ± 36.6	194.5 ± 31.5	196.9 ± 35.0	200.0 ± 42.3	0.020
High-density lipoprotein cholesterol	53.8 ± 11.4	54.8 ± 11.0	54.9 ± 12.0	51.7 ± 11.0	<0.001
Low-density lipoprotein cholesterol	94.6 ± 26.3	92.9 ± 24.4	94.5 ± 24.7	96.3 ± 29.3	0.061
Diabetes, n (%)	409 (20.4)	34 (5.1)	31 (4.6)	344 (51.4)	<0.001
Hypertension, n (%)	837 (41.8)	232 (34.8)	255 (38.2)	350 (52.3)	<0.001
Use of antidiabetic medications, n (%)	251 (12.5)	23 (3.5)	14 (2.1)	214 (32.0)	<0.001
Use of statins, n (%)	13 (0.6)	2 (0.3)	3 (0.4)	8 (1.2)	0.092
Use of ACEIs or ARBs, n (%)	99 (4.9)	26 (3.9)	30 (4.5)	43 (6.4)	0.084
The 2^nd^ visit ba-PWV, cm/sec	1354 ± 190	1329 ± 192	1342 ± 138	1391 ± 184	<0.001
The 3^rd^ visit ba-PWV, cm/sec	1613 ± 286	1572 ± 274	1573 ± 264	1695 ± 302	<0.001
Ba-PWV change**^†^**, cm/sec	259 ± 222	244 ± 206	231 ± 204	303 ± 248	<0.001
Ratio of ba-PWV change**^‡^**, %	19.7 ± 16.8	18.9 ± 16.2	17.8 ± 15.5	22.3 ± 18.3	<0.001
Elevated ba-PWV**^¶^**, n (%)	652 (32.6)	188 (28.2)	175 (26.2)	289 (43.2)	<0.001

Data are baseline characteristics of study participants unless indicated otherwise.

Data are mean ± SD or median (quartile 1-quartile 3) for continuous variables and n (%) for categorical variables.

**^†^**Ba-PWV change was calculated as the 3^rd^ visit ba-PWV-the 2^nd^ visit ba-PWV.

**^‡^**Ratio of ba-PWV change was calculated as ba-PWV change/the 2^nd^ visit ba-PWV.

**^¶^**Elevated ba-PWV was define as ba-PWV ≥ 1705 cm/sec, which was the upper quartile of the 2^nd^ visit ba-PWV.

T, tertile; FPG, fasting plasma glucose; SD, standard deviation; ACEI, angiotensin converting enzyme inhibitor; ARB, angiotensin receptor blocker; ba-PWV, brachial–ankle pulse wave velocity.

### VVV of FPG and Changes of ba-PWV

**Figure 2:**
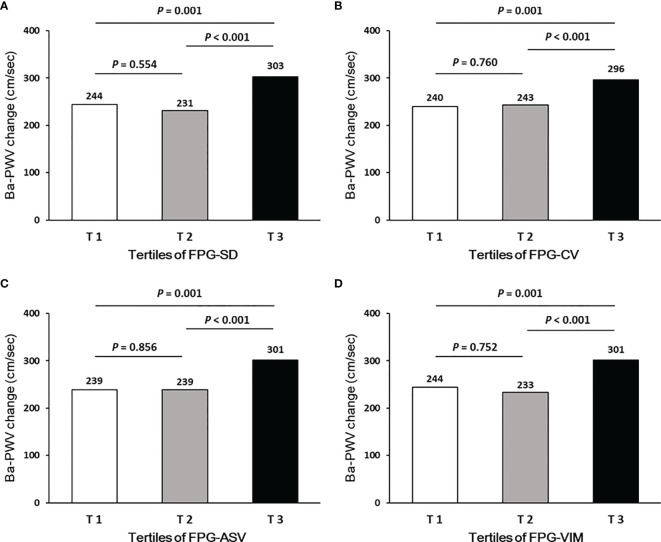
Ba-PWV change according to tertiles of FPG-SD **(A)**, FPG-CV **(B)**, FPG-ASV **(C)** and FPG-VIM **(D)**.

Ba-PWV change, the ratio of ba-PWV change, and the occurrence of an elevated ba-PWV across VVV of FPG tertiles (FPG-SD, FPG-CV, FPG-ASV and FPG-VIM) are shown in **Figures 2** **–** **4**. They were the highest among participants in the highest tertiles, but were similar between the first and second tertiles, respectively.

**Figure 3:**
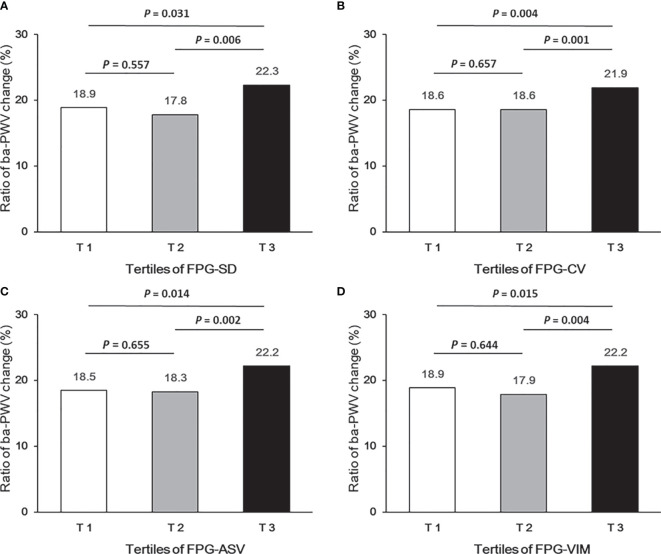
Ratio of ba-PWV change according to tertiles of FPG-SD **(A)**, FPG-CV **(B)**, FPG-ASV **(C)** and FPG-VIM **(D)**.

**Figure 4:**
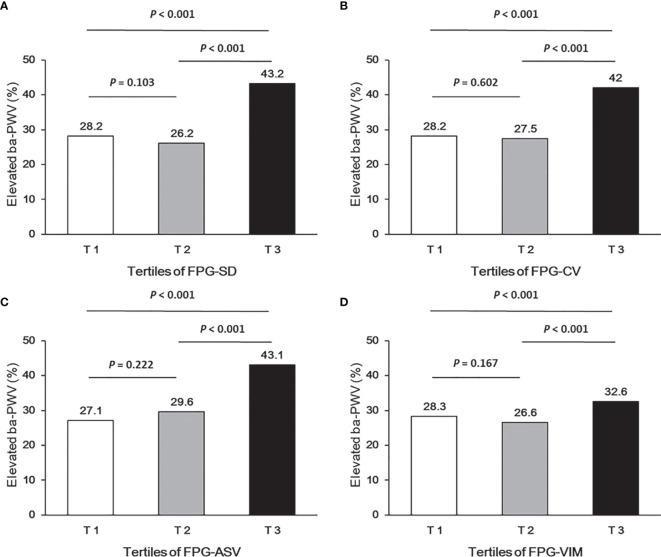
Occurrence of the elevated ba-PWV according to tertiles of FPG-SD **(A)**, FPG-CV **(B)**, FPG-ASV **(C)** and FPG-VIM **(D)**.

By performing multiple linear regression analysis, we found that FPG-SD, FPG-CV, FPG-ASV and FPG-VIM were all independently associated with the ba-PWV changes even after full adjustment for covariates in Model 4 (all *P* values < 0.05; **Table 2**).

**Table 2:** Multiple linear regression analysis of VVV in FPG associated with the changes of ba-PWV.

Measures of variability	Changes of ba-PWV				
		Ba-PWV change (cm/sec)		Ratio of ba-PWV change (%)	
		β ± SE	*P*-value	β ± SE	*P*-value
SD	Model 1	1.705 ± 0.283	<0.001	0.109 ± 0.022	<0.001
	Model 2	1.743 ± 0.352	<0.001	0.111 ± 0.027	<0.001
	Model 3	1.588 ± 0.353	<0.001	0.104 ± 0.027	<0.001
	Model 4	1.223 ± 0.388	0.002	0.085 ± 0.029	0.003
CV	Model 1	2.933 ± 0.483	<0.001	0.188 ± 0.037	<0.001
	Model 2	2.733 ± 0.561	<0.001	0.180 ± 0.043	<0.001
	Model 3	2.470 ± 0.562	<0.001	0.169 ± 0.044	<0.001
	Model 4	2.063 ± 0.563	<0.001	0.147 ± 0.042	<0.001
ASV	Model 1	1.417 ± 0.242	<0.001	0.088 ± 0.019	<0.001
	Model 2	1.389 ± 0.304	<0.001	0.089 ± 0.023	<0.001
	Model 3	1.255 ± 0.304	<0.001	0.083 ± 0.024	<0.001
	Model 4	0.885 ± 0.329	0.007	0.061 ± 0.024	0.012
VIM	Model 1	0.566 ± 0.091	<0.001	0.036 ± 0.007	<0.001
	Model 2	0.545 ± 0.109	<0.001	0.036 ± 0.008	<0.001
	Model 3	0.494 ± 0.109	<0.001	0.033 ± 0.009	<0.001
	Model 4	0.399 ± 0.113	<0.001	0.028 ± 0.008	0.001

Model 1 was adjusted for age and sex.

Model 2 was additionally adjusted for education, current smoking, current drinking, physical activity, diabetes status, use of antidiabetic medications, use of statins and use of ACEIs or ARBs.

Model 3 was additionally adjusted for baseline WC, SBP, log_10_TG, LDL-c, log_10_ (change of TG) and change of LDL-c.

Model 4 was additionally adjusted for average FPG and ba-PWV at the second visit.

VVV, visit-to-visit variability; β, regression coefficient; SE, standard error; SD, the standard deviation; CV, the coefficient of variation; ASV, the average successive variability; VIM, the variability independent of the mean; FPG, fasting plasma glucose; ba-PWV, brachial-ankle pulse wave velocity; ACEI, angiotensin converting enzyme inhibitor; ARB, angiotensin receptor blocker; WC, waist circumference; SBP, systolic blood pressure; log_10_ TG, log_10_ transformed triglycerides; LDL-c, low-density lipoprotein cholesterol.

### VVV of FPG and Elevated ba-PWV

We analyzed the associations between glycemic variability and elevated ba-PWV in multivariate-adjusted logistic regression models (**Table 3**). After full adjustment (model 4), when compared with the lowest tertile, participants in the highest tertile of FPG-SD had a significantly higher risk of developing elevated ba-PWV (OR 1.37 [95% CI 1.01-1.86]). We observed similar results with FPG-CV and FPG-VIM. When compared with the lowest tertile, the highest tertile of FPG-CV yielded an increased risk of elevated ba-PWV development (OR 1.41 [95% CI 1.06-1.88]). The OR and 95% CI for the highest *vs*. the lowest tertile of FPG-VIM for elevated ba-PWV development were 1.39 (1.03-1.87). No significant associations were found for FPG-ASV tertiles and elevated ba-PWV (OR 1.34 [95% CI 0.98-1.82]). No interaction between diabetic status (with and without diabetes) and VVV of FPG in association with elevated ba-PWV was found (all *P* values for interaction > 0.05).

**Table 3:** Logistic regression analysis of VVV in FPG associated with the elevated ba-PWV.

Measures of variability	Estimate of association, OR (95% CI)			
	Model 1	Model 2	Model 3	Model 4
Tertiles of SD				
T1 (0-6.5)	Reference	Reference	Reference	Reference
T2 (6.5-11.9)	0.93 (0.72-1.21)	0.93 (0.72-1.21)	0.86 (0.66-1.13)	0.85 (0.65-1.11)
T3 (11.9-114.2)	2.00 (1.56-2.56)	1.72 (1.30-2.28)	1.49 (1.11-2.00)	1.37 (1.01-1.86)
Tertiles of CV				
T1 (0-7.0)	Reference	Reference	Reference	Reference
T2 (7.0-12.1)	1.04 (0.80-1.34)	1.02 (0.79-1.32)	0.98 (0.75-1.29)	0.97 (0.74-1.26)
T3 (12.1-78.2)	1.94 (1.52-2.49)	1.65 (1.26-2.16)	1.48 (1.12-1.97)	1.41 (1.06-1.88)
Tertiles of ASV				
T1 (0-7.1)	Reference	Reference	Reference	Reference
T2 (7.1-14.0)	1.08 (0.84-1.40)	1.07 (0.82-1.39)	0.99 (0.76-1.30)	0.97 (0.74-1.28)
T3 (14.0-221.1)	2.03 (1.58-2.61)	1.70 (1.28-2.26)	1.46 (1.08-1.96)	1.34 (0.98-1.82)
Tertiles of VIM				
T1 (0-30.8)	Reference	Reference	Reference	Reference
T2 (30.8-54.6)	0.97 (0.75-1.26)	0.97 (0.75-1.26)	0.91 (0.69-1.19)	0.90 (0.69-1.18)
T3 (54.6-409.1)	1.99 (1.55-2.55)	1.70 (1.28-2.24)	1.49 (1.12-2.00)	1.39 (1.03-1.87)

Model 1 was adjusted for age and sex.

Model 2 was additionally adjusted for education, current smoking, current drinking, physical activity, diabetes status, use of antidiabetic medications, use of statins and use of ACEIs or ARBs.

Model 3 was additionally adjusted for baseline WC, SBP, log_10_TG, LDL-c, log_10_ (change of TG) and change of LDL-c.

Model 4 was additionally adjusted for average FPG.

VVV, visit-to-visit variability; OR, odds ratio; CI, confidence internal; SD, the standard deviation; CV, the coefficient of variation; ASV, the average successive variability; VIM, the variability independent of the mean; FPG, fasting plasma glucose; ba-PWV, brachial-ankle pulse wave velocity; ACEI, angiotensin converting enzyme inhibitor; ARB, angiotensin receptor blocker; WC, waist circumference; SBP, systolic blood pressure; log_10_ TG, log_10_ transformed triglycerides; LDL-c, low-density lipoprotein cholesterol.

The four measures of FPG variability were highly correlated (**Supplemental Table 1**). The forward stepwise (conditional) multivariate logistic regression analysis for elevated ba-PWV revealed that FPG-SD remained the only significant factor of the four measures comparing participants in the highest tertile *vs*. the lowest tertile (OR 1.37 [95% CI 1.02-1.84], **Supplemental Table 2**).

## Discussion

In this community-based cohort study, we found that the increased VVV of FPG, measured by FPG-SD, FPG-CV, FPG-ASV and FPG-VIM, was significantly associated with increased arterial stiffness determined by elevated ba-PWV. More importantly, these significant associations were consistent even after further adjustment for the average FPG. To the best of our knowledge, this is the first report investigating an association between VVV of FPG and arterial stiffness in a general population. Our findings suggested that the long-term glycemic variability may play an important role in the development of atherosclerosis at an early stage.

Nowadays, CVD has become the leading cause of morbidity and mortality worldwide (21). In the majority of cases, atherosclerosis is the underlying cause of CVD (22), which is a progressive disease featured as the accumulation of lipids, inflammatory cells, and fibrous elements in the wall of large arteries (23). If patients can be detected with subclinical atherosclerosis in the early stage, their cardiovascular risks would be reduced by early intervention. Arterial stiffness is one of the major manifestations of atherosclerosis (23–25). PWV is the most widely used measure to assess arterial stiffness and has become a useful tool for the early detection and risk stratification of CVD (24). In addition, PWV can be used to predict CVD events, cardiovascular mortality, and all-cause mortality, especially in subjects with a higher baseline cardiovascular risk (8).

Glycemic variability is an assessment of glycemic control which could predict the risk of complications in people with diabetes. Previous studies in animals and humans have mainly focused on the short-term variability in blood glucose, and found that acute and short-term glycemic changes resulted in the excessive production of superoxide and inflammatory cytokines, oxidative stress generation, epigenetic changes and macrophage adhesion to endothelial cells, all of which may promote the development of atherosclerosis (26–29). However, atherosclerosis is a chronic process which goes on for years or decades and the long-term variability of FPG might be more important than the short-term glycemic change in association and prediction of atherosclerosis. Studies which assessed the long-term glycemic variability are accumulating in people with or without diabetes (19, 30–34). Most of them concluded that the long-term glycemic variability may be a predictor of all-cause mortality (19, 30, 31), fatal or non-fatal CVD (30–32, 34). The underlying pathological mechanism is considered to be complicated and has not been understood fully. The “metabolic memory” phenomenon is one of the possible mechanisms (35, 36). Unlike these previous studies mentioned above, our study mainly focused on the association of long-term fasting glucose variability with subclinical atherosclerosis.

Long-term glycemic variability is an index that has been frequently reported in recent years, but there is no standardized measure for it (5). HbA1c is a time averaged mean glycemia level, while FPG level is more likely to capture acute fluctuation in glucose level owing to lifestyle episodes or irregular eating. Thus, FPG might be a more sensitive indicator than HbA1c for extra variation of glucose. Similarly, visit-to-visit FPG variability and HbA1c variability are markers for glucose variability that capture different aspects. Although many previous studies assessed the glycemic oscillation by HbA1c variability which has been found to be significantly associated with outcomes, the seasonal fluctuations in HbA1c should be considered (5). HbA1c evaluated on a visit-to-visit basis may be underestimated or overestimated depending on the timing of blood sampling. Alternatively, FPG variability may have captured more accurately hypoglycemic episodes than HbA1c variability. There is an intriguing finding in a previous study that FPG variability may present a stronger relationship than HbA1c variability, especially for cardiovascular and all-cause mortality (34). The differences between these four measures in terms of magnitude and significance of the observed associations with outcomes suggest that these measures probably capture different aspects of variability. In the current study, we used FPG-SD, FPG-CV, FPG-ASV and FPG-VIM as the four measures of the long-term glycemic variability and found that VVV of FPG was independently associated with arterial stiffness. Our results also suggested that FPG-SD is possibly a more robust measure, which was more closely related to arterial stiffness among these four measures.

The current study adds evidence of association between long-term FPG variability and arterial stiffness. However, our study has several limitations. First, we examined the association between VVV of FPG which was calculated by the FPG levels at the 3 visits and arterial stiffness which was determined by the 2^nd^ and 3^rd^ visits ba-PWV levels. We did not have ba-PWV data at the 1^st^ visit. It would be better if the 1^st^ visit ba-PWV were included in the analyses to indicate the arterial stiffness. A prospective investigation with the assessment of ba-PWV changes after the 3^rd^ visit is required in order to better elucidate the role that long-term-glycemic variability plays in the process of arterial stiffness. Second, we did not have data on HbA1c, the glycemic marker indicating the mean glycemia. Long-term variability in HbA1c may significantly affect VVV of FPG and provide a different and complementary perspective on the association of glycemic variability with outcomes. Third, the small sample size of this study limited the possibility to stratify the participants based on diabetic status (with and without diabetes). In addition, the study participants were middle-aged and elderly Chinese adults recruited from suburban Shanghai, so the results may not be generalizable to younger individuals or other ethnicities.

In conclusion, greater VVV of FPG is significantly associated with the increased arterial stiffness, independently of the average FPG. There is likely to be a common pathophysiological link between long-term FPG variability and arterial stiffness. Based on these results, our findings add to the growing body of evidence for the prognostic value of long-term glucose variability and underscore the importance of long-term stable glucose control. Early initiation of glycemic variability management is likely to be beneficial to the early prevention of atherosclerosis. More studies are needed to further elucidate the linkage mechanisms and the importance of the long-term glycemic variability in provoking early macrovascular diseases in diverse populations.

## Data Availability Statement

The original contributions presented in the study are included in the article/**Supplementary Material**. Further inquiries can be directed to the corresponding authors.

## Ethics Statement

The studies involving human participants were reviewed and approved by The Institutional Review Board of Rui-Jin Hospital, Shanghai Jiao Tong University School of Medicine. The patients/participants provided their written informed consent to participate in this study.

## Author Contributions

YZ, SWu, YX, and YC had access to all data and take responsibility for its integrity and analysis. YZ and YX conceived the hypotheses and analyses. YZ drafted the paper. YZ and SWu provided statistical analysis. YZ, SWu, and JZ collected the data. YX and YC revised the manuscript. ML, TW, MX, JL, SWa, YB, WW and GN refined interpretation and the final manuscript. All authors contributed to the article and approved the submitted version.

## Funding

This work was funded by the Ministry of Science and Technology of the People’s Republic of China (2017YFC1310700, 2016YFC1304904 and 2016YFC1305600), the National Natural Science Foundation of China (81870560, 81870604 and 8151128019), National Science and Technology Major Project for “Significant New Drugs Development” (2017ZX09304007) and Innovative Research Team of High-level Local Universities in Shanghai. Yuhong Chen was supported by the Three-Year Plan for Promoting Clinical Skills and Innovation in Municipal Hospitals of Shanghai Shenkang Hospital Development Center (16CR4020A). YZ was supported by the Scientific Research Project of Shanghai Health and Family Planning Commission (20174Y0100) and the Talent Training Program Foundation for Youths from Rui-Jin Hospital North (2017RCPY-B10).

## Conflict of Interest

The authors declare that the research was conducted in the absence of any commercial or financial relationships that could be construed as a potential conflict of interest.

The reviewer LL declared a shared affiliation, with no collaboration, with the authors to the handling editor at the time of the review.

## Publisher’s Note

All claims expressed in this article are solely those of the authors and do not necessarily represent those of their affiliated organizations, or those of the publisher, the editors and the reviewers. Any product that may be evaluated in this article, or claim that may be made by its manufacturer, is not guaranteed or endorsed by the publisher.
