# Supplementary material for: Long-Term Glycemic Variability Is Associated With Arterial Stiffness in Chinese Adults
Source: Front Endocrinol (Lausanne). 2021 Sep 16;12:711540. doi: 10.3389/fendo.2021.711540 (PMC8481863; doi:10.3389/fendo.2021.711540)
Supplement: Supplementary file 1 [file Table_1.docx]

**Supplementary Data**

**Supplemental Table 1.** *Pearson’s* correlation coefficient between the four measures of FPG variability

|  | FPG-SD | FPG-CV | FPG-ASV | FPG-VIM |
| --- | --- | --- | --- | --- |
| FPG-SD | 1 | - | - | - |
| FPG-CV | 0.946 | 1 | - | - |
| FPG-ASV | 0.955 | 0.901 | 1 | - |
| FPG-VIM | 0.978 | 0.993 | 0.932 | 1 |

VVV, visit-to-visit variability; FPG, fasting plasma glucose; SD, the standard deviation; CV, the coefficient of variation; ASV, the average successive variability; VIM, the variability independent of the mean.

**Supplemental Table 2.** Multivariate forward stepwise (conditional) logistic regression analysis of related factors of elevated ba-PWV

| Variable | B | SE | Wald | df | OR (95% CI) | *P* value |
| --- | --- | --- | --- | --- | --- | --- |
| Constant | -11.660 | 0.660 | 311.919 | 1 | / | ＜0.001 |
| Age | 0.091 | 0.007 | 162.730 | 1 | 1.10 (1.08-1.11) | ＜0.001 |
| Sex | 0.403 | 0.114 | 12.559 | 1 | 1.50 (1.20-1.87) | ＜0.001 |
| SBP | 0.031 | 0.003 | 98.653 | 1 | 1.03 (1.03-1.04) | ＜0.001 |
| LDL-c | 0.004 | 0.002 | 3.926 | 1 | 1.00 (1.00-1.01) | 0.048 |
| Average FPG | 0.006 | 0.002 | 9.492 | 1 | 1.01 (1.00-1.01) | 0.002 |
| Tertiles of FPG-SD | | | | | | 0.006 |
| T1 | / | / | 10.127 | 2 | Reference | / |
| T2 | -0.157 | 0.138 | 1.297 | 1 | 0.86 (0.65-1.12) | 0.255 |
| T3 | 0.317 | 0.151 | 4.406 | 1 | 1.37 (1.02-1.84) | 0.036 |

Variables added into the model included age, sex, education, current smoking, current drinking, physical activity, diabetes status, use of antidiabetic medications, use of statins, use of ACEIs or ARBs, baseline WC, SBP, log_10_TG, LDL-c, log_10_ (change of TG) and change of LDL-c, average FPG, FPG-SD, FPG-CV, FPG-ASV and FPG-VIM.

Ba-PWV, brachial-ankle pulse wave velocity; SE, Standard error; OR, odds ratio; CI, confidence internal; SBP, Systolic blood pressure; LDL-c, Low-density lipoprotein cholesterol; FPG, fasting plasma glucose; SD, the standard deviation. FPG, fasting plasma glucose; ACEI, angiotensin converting enzyme inhibitor; ARB, angiotensin receptor blocker; WC, waist circumference; log_10_ TG, log_10_ transformed triglycerides; LDL-c, low-density lipoprotein cholesterol; CV, the coefficient of variation; ASV, the average successive variability; VIM, the variability independent of the mean.
